# Plate Clearing and Body Mass Index: A Meta‐Analysis

**DOI:** 10.1002/osp4.70118

**Published:** 2026-01-20

**Authors:** Adrian Meule, Lisa Dietlmeier, David R. Kolar

**Affiliations:** ^1^ Department of Psychology University of Regensburg Regensburg Germany

**Keywords:** body mass index, obesity, overweight, plate cleaning, plate clearing

## Abstract

**Background:**

Current food environments are characterized by larger food portions, which contribute to higher food consumption. Thus, habitually finishing meals by eating the entire portion (so‐called plate clearing or plate cleaning) may lead to weight gain. However, findings have been mixed: some studies reported small, positive associations between self‐reported plate clearing tendencies and body mass index, but other studies did not find a relationship or even reported a negative association.

**Methods:**

The current study performed a meta‐analysis on the correlation between plate clearing tendencies and body mass index.

**Results:**

The pooled effect based on 22 samples was *r* = 0.04 (95% CI [−0.02, 0.10]), indicating no relationship between plate clearing and body mass index. A meta‐regression indicated that the percentage of women as well as the type of self‐report measure moderated the effect, suggesting that there might be a small, positive relationship between plate clearing and body mass index in men and when the Plate Clearing Tendency Scale was used.

**Conclusion:**

This meta‐analysis does not indicate that habitual plate clearing relates to a higher body weight in general. While self‐report biases cannot be excluded based on the current study, the absence of an observed association highlights the need for further exploration into why this relationship is not evident.

## 1 Introduction

Current food environments are characterized by larger food portions [1], which contribute to higher food consumption [2, 3]. Thus, habitually finishing meals by eating the entire portion (so‐called plate clearing or plate cleaning) may lead to weight gain. However, findings on the relationship between plate clearing and body weight have been mixed. For example, two early studies from the 1970s conducted in natural settings suggested that women with overweight tended to clear their plates more than women with normal weight did [4, 5], but this finding could not be replicated in a later study, in which food intake was observed in a university cafeteria and in which women with overweight, in fact, left more food on their plates than women with normal weight [6].

More recent studies often measured plate clearing tendencies through self‐reporting, usually with the Plate Clearing Tendency Scale (PCTS, ref. [7]). The PCTS is a brief, internally reliable measure with five items (e.g., “I normally finish eating when my plate is empty.”) that measures plate clearing tendencies as a unidimensional construct [7, 8, 9]. Responses are recorded on a 5‐point scale ranging from 1 = *strongly disagree* to 5 = *strongly agree*. However, while the PCTS appears to be a psychometrically sound tool, it seems that it also did not produce consistent evidence for a relationship between plate clearing and body weight: some studies reported a small, positive association between PCTS scores and body mass index (BMI, based on self‐reported body height and weight; e.g., refs. [7, 10]), whereas other studies did not find a relationship (based on either self‐reported or measured body height and weight; refs. [8, 9, 11, 12]).

Because of these inconsistent findings, we conducted a meta‐analysis on the relationship between plate clearing and BMI according to a preregistered protocol (https://doi.org/10.17605/OSF.IO/AU7M2). Given that some studies reported a small positive association while others found no relationship, we hypothesized that the pooled effect across studies would indicate that plate clearing is unrelated or, at most, weakly positively related to BMI (*r* = 0.1–0.2). As outlined in the preregistration, we also tested possible moderators of the relationship between plate clearing and BMI with meta‐regression models if there were at least 10 studies available (for continuous moderators) or at least five studies available for each category (for categorical moderators). Our general approach was to include all moderators for which sufficient data were available across most studies, such as publication year, samples' mean age and mean BMI, percentage of females, country, and type of self‐report measure. Other potentially relevant moderators—such as study type (e.g., online study vs. laboratory or field study)—could not be examined because of an insufficient number of studies. We did not have directed hypotheses about the nature of possible moderation effects.

## 2 Methods

### 2.1 Literature Search, Screening, and Study Selection

The literature search was conducted on November 25, 2024 using Google Scholar (https://scholar.google.com) with the following keywords and Boolean operators:

((“plate clearing”) OR (“plate cleaning”)) AND ((“body mass index”) OR (“bmi”)).

To ensure comprehensive retrieval, no filters were applied based on publication date or document category, so all materials indexed by Google Scholar (including journal articles, conference papers, theses, and other gray literature) were captured. The search was conducted exclusively via Google Scholar, given its demonstrated breadth of coverage, usually including materials indexed by curated platforms such as Web of Science [13, 14]. The collected search results were imported into Rayyan (https://www.rayyan.ai) to facilitate duplicate removal and streamline the screening workflow. All screening decisions (including removal of duplicates before screening) were made manually by two independent reviewers within Rayyan with disagreements addressed through discussion; no automated procedures provided by the software were used. To enhance coverage, reference lists of the included studies were reviewed to identify additional eligible publications. Studies were included if they reported BMI and used a self‐report measure of plate clearing or measured laboratory food intake in a way that allowed to clearly categorize participants as having finished their portion or not. Based on a reviewer's suggestion, we conducted an updated literature search on December 8, 2025, to capture studies published in 2025. This search did not identify any additional relevant studies.

### 2.2 Data Extraction

The following information was retrieved from the included reports: publication year, sample size, correlation coefficient between the plate clearing measure and BMI, type of plate clearing measure, the samples' mean age, mean BMI as well as percentage of females, and country, in which the study was conducted. We also planned to code the age group (children, adolescents, adults, mixed) and BMI category (underweight, normal weight, overweight, obesity, mixed) of each sample but as all studies investigated adult samples with mixed BMI categories, this information was not used for meta‐regression analyses. When studies measured the relevant data but did not report it, we reached out to the corresponding authors on two occasions within a 4‐week period. Studies were excluded if we could not obtain a correlation coefficient for the relationship between plate clearing and BMI. If a correlation coefficient was reported in a paper, we used this one (irrespective whether authors specified which kind of correlation coefficient it was). If we were able to compute a correlation coefficient ourselves (either because the data are publicly available or the authors sent us the data) or asked the authors to send a correlation coefficient, we computed or requested Pearson's *r* (note that if there were laboratory studies that measured plate clearing as a binary variable, Pearson's *r* is equivalent to the point‐biserial correlation coefficient).

### 2.3 Data Analyses

Analyses were run with R version 4.5.0 in RStudio version 2025.05.0 and with JASP version 0.19.3. Meta‐analysis was conducted with the *meta* package version 8.1–0 by pooling correlation coefficients using a random‐effects model with the *metacor* function, which first performs Fisher's *z*‐transformation and then applies the generic inverse variance method for meta‐analytic pooling. Knapp–Hartung adjustments were applied to calculate the confidence interval around the pooled effect. Heterogeneity was examined with *τ* ^2^, *I* ^2^ and prediction intervals. Restricted maximum likelihood was used as an estimator for calculating the heterogeneity variance *τ* ^2^. Outlier and influence diagnostics were conducted using the *dmetar* package version 0.1.0. The *find.outliers* function was employed to identify outlying studies and subsequently re‐estimate the model excluding these data points. Additionally, leave‐one‐out analysis using the *InfluenceAnalysis* function was conducted, whereby the model was iteratively re‐run with each study removed in turn to assess its individual impact. Meta‐regressions were performed to examine moderators of the relationship between plate clearing and BMI with the *meta* package's *metareg* function. Separate models were run for each of the following moderator variables: publication year, mean age, mean BMI, country (UK vs. other), percentage of females, and type of self‐report measure.

Egger et al.’s [15] regression test was run using the *meta* package's *metabias* function to examine asymmetry in the funnel plot. We also examined effect estimates adjusted for possible publication bias using the *meta* package's *trimfill* function for applying Duval & Tweedie's [16] trim‐and‐fill method and additionally used the *limitmeta* function and *copas* function from the *metasens* package version 1.5–3 for applying Rücker et al.’s [17] limit meta‐analysis method and Copas' [18] selection models. Moreover, we derived bias‐corrected estimates with the Precision‐Effect Test–Precision‐Effect Estimate with Standard Error (PET–PEESE; [19]) and the Weighted Average of the Adequately Powered effect size using Weighted Least Squares (WAAP–WLS; [20]) using JASP's meta‐analysis module. Comprehensive explanations of the methods used to address publication bias can be found in external sources to which we direct interested readers [21, 22, 23]. The data, R code, and JASP output file for reproducing all results can be accessed at https://doi.org/10.17605/OSF.IO/D4ZH3.

## 3 Results

### 3.1 Summary of Included Studies

**FIGURE 1:**
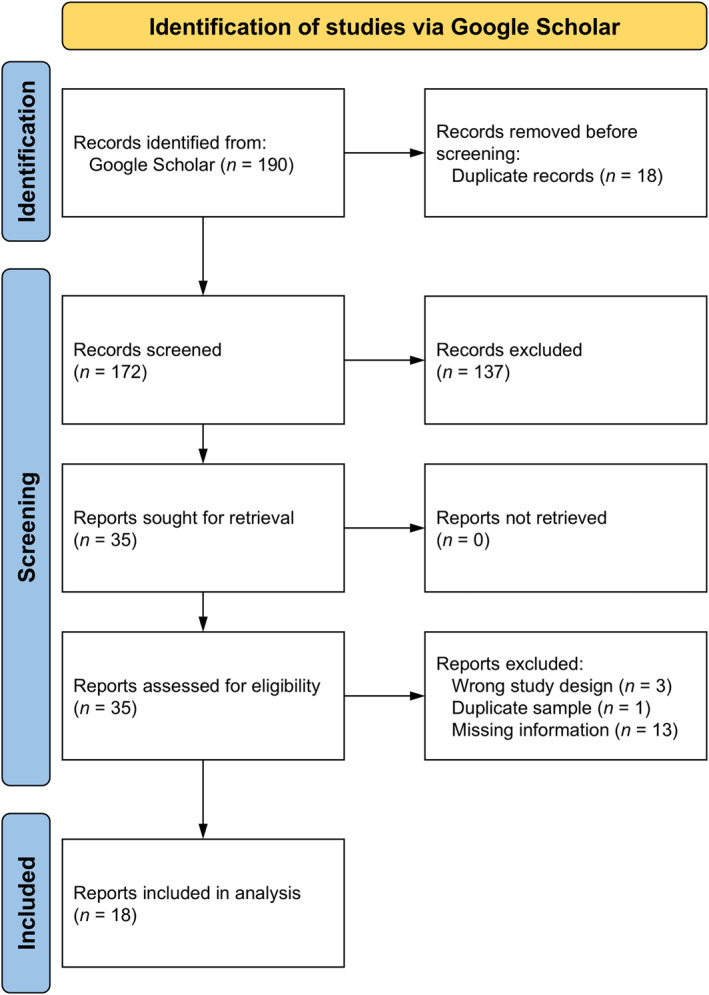
Flowchart illustrating the screening and study selection process.

Out of 190 records retrieved through the literature search, 18 were ultimately included in the meta‐analysis (Figure 1).^1^ Data from 22 samples described in these 18 reports were used for meta‐analysis (Table 1). Most studies were conducted in the UK (*k* = 13) and nine studies were conducted in other countries (the Netherlands, Germany, Turkey, Japan, Singapore, USA). As a measure of plate clearing, 16 studies used the PCTS, five studies used other self‐reports (i.e., single questions such as “How often do you leave food on your plate?”; ref. [27]), and only one study was based on laboratory food intake. Note that we originally had included another study that measured laboratory food intake (Study 3 in ref. [11]) for which the correlation coefficient for the relationship between plate clearing (that we defined as finishing at least 95% of the portion, which resulted in two groups of *n* = 30 plate clearers and *n* = 98 non‐plate clearers) and BMI was *r* = 0.06. However, we decided to remove this from the data set as we had already included this study using the correlation coefficient for the relationship between PCTS scores and BMI, which would otherwise have resulted in a duplicate sample. Information on the samples' mean age, mean BMI, and percentage of females was missing for one study [35], which is why meta‐regression models using these variables are based on 21 samples.

**TABLE 1:** Details of included studies.

Study	*N*	*r*	Country	Type of measure	Mean age (years)	Proportion of females (%)	Mean body mass index (kg/m^2^)
Best & Papies, 2019 [24]	511	0.04	UK	PCTS	37.8	67	27.5
Cheon et al., 2019 [25]	33	0.20	Singapore	Laboratory food intake^a^	22.2	48	21.3
Fay et al., 2011 [26]	764	0.03	UK	Other self‐report	25.6	78	22.8
Hinton et al., 2024 [27]	846	−0.07	UK	Other self‐report	33.0	69	24.6
Kawasaki et al., 2024 [28]	1800	0.003	Japan	Other self‐report	40.2	50	21.8
Langfield et al., 2023 [29]							
Study 1	50	−0.17	UK	PCTS	42.3	100	25.8
Study 2	46	0.12	UK	PCTS	51.6	100	28.0
Langfield et al., 2023 [30]	77	−0.16	UK	PCTS	41.7	51	30.7
Marchiori & Papies, 2014 [31]	110	−0.2	The Netherlands	Other self‐report	20.9	71	22.3
Nill & Meule, 2022 [8]	207	0.05	Germany	PCTS	29.6	76	23.9
Robinson et al., 2015 [7]	993	0.15	USA	PCTS	31	40	26.5
Robinson & Hardman, 2016 [10]	385	0.15	UK	PCTS	22.8	76	23.6
Robinson & Haynes, 2021 [32]	116	−0.14	UK	PCTS	31.1	50	26.8
Robinson et al., 2015 [33]							
Study 1	124	0.25	USA	PCTS	30	0	27.1
Study 2	117	0.33	USA	PCTS	29.7	0	26.4
Study 3	88	−0.03	UK	PCTS	33.1	50	25.3
Şarahman Kahraman et al., 2024 [9]	333	−0.02	Turkey	PCTS	32.4	67	24.2
Sheen, 2020 [34]	159	0.21	UK	PCTS	25.8	50	24.2
Sheen et al., 2018 [12]	88	0.04	UK	PCTS	25.4	100	22.4
Sheen et al., 2020 [11]							
Study 2	212	0.06	UK	PCTS	25.4	77	24.8
Study 3	128	0.14	UK	PCTS	22.7	66	23.9
Velez et al., 2017 [35]	135	−0.10	USA	Other self‐report	NA	NA	NA

Abbreviations: NA = not available, PCTS = plate clearing tendency scale, UK = United Kingdom, USA = United States of America.

^a^ Based on categorizing the sample into 28 plate clearers and 5 non‐plate clearers and computing the point‐biserial correlation coefficient between this group variable (0 = no, 1 = yes) and body mass index.

### 3.2 Pooled Effect Size, Heterogeneity, Outliers, and Influential Studies

**FIGURE 2:**
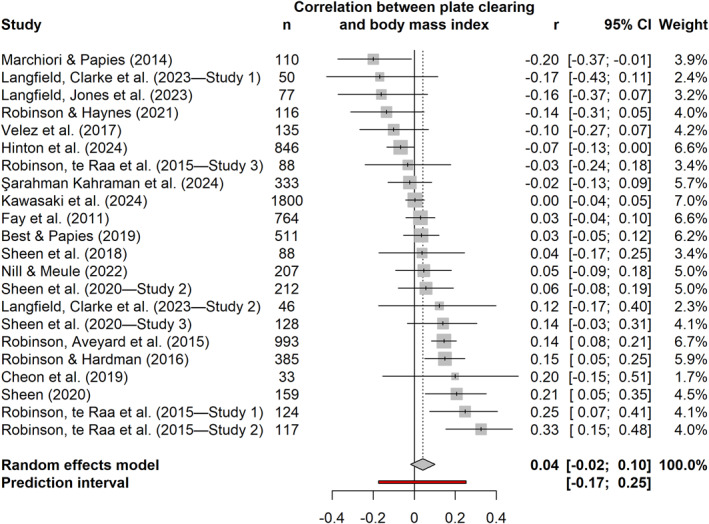
Forest plot for the meta‐analysis of the relationship between plate clearing and body mass index. Gray squares depict individual study effect estimates, scaled according to their statistical weight. Horizontal black lines extending from each square represent the associated 95% confidence intervals. The pooled estimate is visualized by the midpoint of the gray diamond, with its span indicating the 95% confidence interval. A red line illustrates the 95% prediction interval, capturing the expected dispersion of effect sizes in future analogous research.

Based on 22 samples, the pooled effect for the relationship between plate clearing and BMI was *r* = 0.04 (95% CI [−0.02, 0.10], *p* = 0.163; Figure 2). Between‐study heterogeneity was moderate (*τ* ^2^ = 0.01, 95% CI [0.004, 0.03]; *I* ^2^ = 69.9%, 95% CI [53.6, 80.5]; prediction interval [−0.17, 0.25]). One outlier was identified (Study 2 in ref. [33]) but its exclusion produced a similar estimate of *r* = 0.03 (95% CI [−0.02, 0.08], *p* = 0.263). Furthermore, all effect estimates in the leave‐one‐out analysis ranged between *r* = 0.03 and *r* = 0.05, indicating no influential studies.

### 3.3 Meta‐Regressions

**FIGURE 3:**
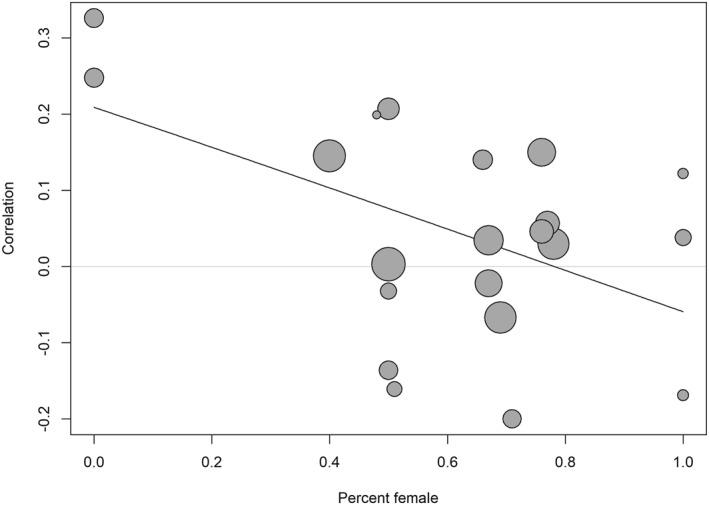
Bubble plot visualizing the meta‐regression effect of a lower percentage of females relating to a larger correlation between plate clearing and body mass index. Larger bubbles indicate larger weights.

**FIGURE 4:**
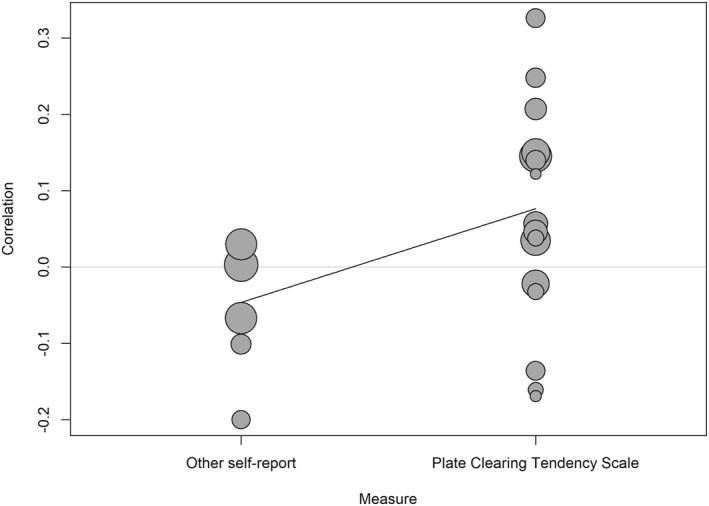
Bubble plot visualizing the meta‐regression effect of studies that used the Plate Clearing Tendency Scale relating to a larger correlation between plate clearing and body mass index than studies that used other self‐reports. Larger bubbles indicate larger weights.

Publication year (*b* = −0.01, SE = 0.01, 95% CI [−0.03, 0.004], *p* = 0.155), mean age (*b* = −0.01, SE = 0.004, 95% CI [−0.01, 0.004], *p* = 0.248), mean BMI (*b* = 0.002, SE = 0.01, 95% CI [−0.03, 0.03], *p* = 0.912), and country (*b* = −0.04, SE = 0.06, 95% CI [−0.16, 0.09], *p* = 0.545) did not moderate the effect. However, the percentage of females moderated the effect (*b* = −0.27, SE = 0.11, 95% CI [−0.50, −0.05], *p* = 0.021) such that the relationship between plate clearing and BMI was lower with a higher percentage of females (Figure 3). To facilitate interpretation, we ran the meta‐analysis with a subset of four studies in which the percentage of females was lower than 50%, which yielded a small but significant, positive relationship between plate clearing and BMI (*r* = 0.21, 95% CI [0.07, 0.34], *p* = 0.017). In a subset of 13 studies in which the percentage of females was higher than 50%, the relationship between plate clearing and BMI was not significant (*r* = 0.01, 95% CI [−0.05, 0.07], *p* = 0.672). The type of self‐report measure also moderated the effect (*b* = 0.12, SE = 0.06, 95% CI [0.004, 0.24], *p* = 0.044) such that the relationship between plate clearing and BMI was higher in studies that used the PCTS than in studies that used other self‐reports (Figure 4). To facilitate interpretation, we ran the meta‐analysis with a subset of 16 studies in which the PCTS was used, which yielded a small but significant, positive relationship between plate clearing and BMI (*r* = 0.08, 95% CI [0.004, 0.15], *p* = 0.041). In a subset of five studies in which other self‐reports were used, the relationship between plate clearing and BMI was not significant (*r* = −0.03, 95% CI [−0.13, 0.06], *p* = 0.254).

### 3.4 Publication Bias

**FIGURE 5:**
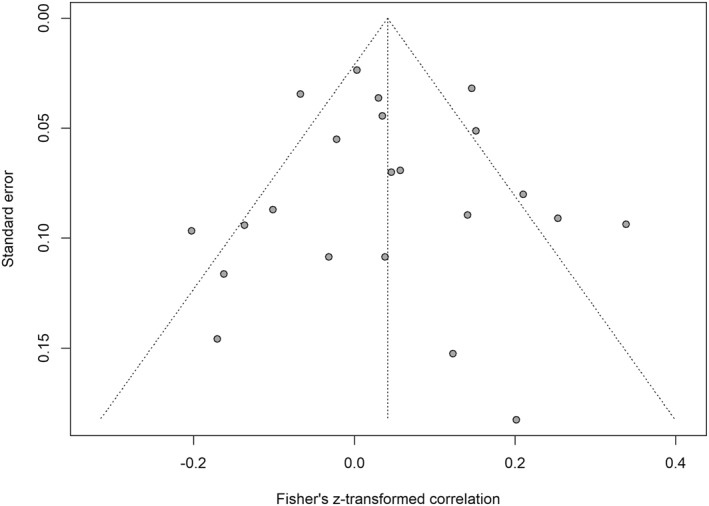
Funnel plot visualizing each study's effect size and standard error. Egger's regression test indicated no asymmetry in this plot, suggesting no publication bias.

Egger et al.’s regression test detected no funnel plot asymmetry (*b* = 0.19, SE = 0.78, 95% CI [−1.34, 1.72], *p* = 0.810; Figure 5). The trim‐and‐fill analysis did not add any studies. The limit meta‐analysis produced an adjusted estimate of *r* = 0.05 (95% CI [−0.04, 0.13], *p* = 0.264). Copas selection models produced an adjusted estimate of *r* = 0.04 (95% CI [−0.01, 0.09], *p* = 0.118). The PET test of publication bias was not significant (*b* = 0.19, SE = 0.78, 95% CI [−1.33, 1.71], *p* = 0.810) and the estimates were *r* = 0.03 (95% CI [−0.06, 0.11], *p* = 0.524) for the PET model and *r* = 0.04 (95% CI [−0.02, 0.09], *p* = 0.210) for the PEESE model. The WAAP model could not be estimated as no studies were deemed as adequately powered, probably due to the overall effect being close to zero.

## 4 Discussion

The current meta‐analysis does not indicate that plate clearing relates to higher body weight. Specifically, the pooled estimate for the correlation between plate clearing and BMI was close to and did not differ from zero. Further analyses suggested that this finding was not affected by outliers or other influential studies and there was no indication of publication bias. We speculate that there are at least two possible explanations for this.

First, one reason for the absent relationship between plate clearing and BMI may be ceiling effects, as plate clearing is quite common. For example, 91% of participants indicated to have eaten their last meal in entirety in the study by Fay and colleagues [26], and similarly, at least 90% of self‐selected food portions were consumed in 86% of meals in a study by Hinton and colleagues [36]. However, when inspecting the means, standard deviations, and ranges of PCTS scores, it seems that there was indeed quite some variation in plate clearing tendencies in the studies included in this meta‐analysis (e.g., refs. [8, 9]). Thus, ceiling effects as an explanation for the absence of a relationship between plate clearing and BMI in the current meta‐analysis may be rather unlikely.

Second, most plate clearing appears to be a pre‐planned behavior rather than a spontaneous act of overeating. For example, the study by Fay et al. [26] found that 92% of all plate‐cleared meals were planned. It is plausible that such tendencies are established early in life, as higher plate clearing in adulthood has consistently been linked to parental encouragement to finish meals during childhood (e.g., refs. [8, 10]). Thus, it may be speculated that persons who habitually clear their plates have learned to adjust portion sizes in advance, thereby minimizing the risk of caloric overconsumption, which is why they do not have a higher body weight. Yet, people usually use portion size as a heuristic for appropriateness, that is, automatically accept the portion that they are served as being of an appropriate size and eat accordingly [37]. Thus, future research could examine if or under which circumstances plate clearing reflects conscious portion adjustment. For example, factors such as the frequency of eating meals outside the home where portion sizes cannot be self‐adjusted may moderate whether regular plate clearing contributes to weight gain.

Although there was no overall relationship between plate clearing and BMI, two moderators were found: type of self‐report measure and sex. Specifically, there was a small, positive association in studies that used the PCTS and no association in samples that used other self‐report measures, which may indicate that using the PCTS measures plate clearing tendencies more reliably than when using single questions. Moreover, there was a small, positive association in samples that predominantly consisted of males and no association in samples that predominantly consisted of females. There is some evidence for sex differences such that men tend to clear their plate more than women do [26, 36], but such sex differences have not consistently been found with the PCTS (e.g., ref. [8]). However, the moderating effect of sex is in line with another finding that suggested that plate clearing may indeed relate to higher body weight but only in certain subgroups. Specifically, higher PCTS scores were only related to higher BMI in a subgroup of unsuccessful dieters but not in successful dieters or non‐dieters [8]. The current meta‐analysis suggests that another such subgroup may be men, but as this was based on a handful of studies only, this finding requires replication in future studies.

The current meta‐analysis was mostly based on self‐reported plate clearing tendencies. In fact, only one study that measured food intake in the laboratory was included. There are several reasons for this although we did indeed find several studies that were possibly relevant to consider. First, the amount of food in laboratory food intake studies is usually intentionally chosen to be unfinishable or not. Thus, some studies had none or only few plate clearers or none‐clearers, and these studies could therefore not be included. Second, some studies did not indicate how many participants finished their portion and the authors did not respond to our request or were not able to determine whether a person was a plate clearer or not. Third, some studies used within‐subject designs with more than one meal, thus making a clear categorization into plate clearers and none‐clearers unfeasible.

As the overall effect for the relationship between plate clearing did not differ from zero and the only laboratory‐based estimate that was included in this analysis was *r* = 0.20 (see Table 1) and the one laboratory‐based estimate that we excluded was *r* = 0.06 (see Section 3.1), it seems that results would probably not look much different if more studies using laboratory food intake were included. Moreover, while self‐report measures can be biased due to demand effects or social desirability, laboratory food intake can be biased by the very same effects as well. Specifically, participants may intentionally finish a meal when they think this is expected by the experimenter or may intentionally not finish a meal due to feelings of shame [38, 39]. Likewise, it has been found that participants eat less in such situations when they are aware that their food intake is being observed [40]. Thus, including more studies that measured whether persons finished their meals or not in the laboratory may not necessarily lead to more reliable or precise estimates of the relationship between plate clearing and BMI.

Another limitation of the present meta‐analysis is that all included studies relied on BMI, which—although it is generally highly correlated with percent body fat [41]—is a relatively crude proxy for percent body fat in subgroups of persons (e.g., children and adolescents, athletes, older adults, certain ethnic groups). Future research incorporating more precise assessments of body composition could provide clearer insights into the relationship between plate clearing tendencies and adiposity. Additionally, findings were based on cross‐sectional designs, which cannot capture the dynamic nature of plate clearing behavior across multiple meals or over time. Longitudinal studies with repeated measures in daily life would be valuable to better understand how plate clearing tendencies relate to weight outcomes and to identify potential mediators and moderators of this relationship.

In conclusion, this meta‐analysis does not indicate that habitual plate clearing relates to a higher body weight in general. Further research in people's daily life over multiple mealtime instances is needed to understand when and how habitual plate clearing may impact weight gain.

## Author Contributions

**Adrian Meule:** conceptualization, formal analysis, supervision, validation, visualization, writing – original draft. **Lisa Dietlmeier:** data curation, formal analysis, investigation, methodology, writing – review and editing. **David R. Kolar:** resources, supervision, writing – review and editing.

## Funding

The authors have nothing to report.

## Ethics Statement

The authors have nothing to report.

## Conflicts of Interest

The authors declare no conflicts of interest.

## Data Availability

Preregistration details, along with the data and code necessary for reproducing all results, are available at https://doi.org/10.17605/OSF.IO/D4ZH3.

## Endnotes

^1^
For reference, conducting a similar search in Web of Science on the same day only yielded three hits, all of which were included in the Google Scholar search results as well.
